# Low efficacy of vaccination against serogroup B meningococci in patients with atypical hemolytic uremic syndrome

**DOI:** 10.1042/BSR20200177

**Published:** 2020-03-25

**Authors:** Nils Mülling, Hana Rohn, Ulrich Vogel, Heike Claus, Benjamin Wilde, Ute Eisenberger, Andreas Kribben, Oliver Witzke, Anja Gäckler

**Affiliations:** 1 Department of Nephrology, University Hospital Essen, University Duisburg-Essen, Essen, Germany; 2 Department of Infectious Diseases, University Hospital Essen, University Duisburg-Essen, Essen, Germany; 3 Institute for Hygiene and Microbiology, National Reference Laboratory for Meningococci and Haemophilus influenzae, University of Würzburg, Würzburg, Germany

**Keywords:** atypical hemolytic uremic syndrome, immunosuppression, meningococcal serogroup B, serum bactericidal antibody titers, vaccination

## Abstract

*Background:* The C5 complement inhibitor eculizumab is first-line treatment in atypical hemolytic uremic syndrome (aHUS) going along with a highly increased risk of meningococcal infections. Serogroup B meningococci (MenB) are the most frequently encountered cause for meningococcal infections in Europe. Efficacy of the protein-based MenB-vaccine Bexsero in aHUS has not been determined and testing is only possible in patients off-treatment with eculizumab as a human complement source is required.

*Methods:* Patients with aHUS were vaccinated with two doses of the protein-based MenB-vaccine Bexsero. Serum bactericidal antibody (SBA) titers against factor H binding protein (fHbp) of MenB were determined in 14 patients with aHUS off-treatment with eculizumab.

*Results:* Only 50% of patients showed protective human serum bactericidal antibody (hSBA) titers (≥1:4) against MenB following two vaccinations. Bactericidal antibody titers were relatively low (≤1:8) in three of seven patients with protective titers. While 71% of patients were on immunosuppressive treatment for either thrombotic microangiopathy or renal transplantation at either first or second vaccination, all four patients not receiving any immunosuppressive treatment showed protective bactericidal antibody response. Time between second vaccination and titer measurement was not significantly different between patients with protective titers compared with those with non-protective titers, while time between first and second vaccination was significantly longer in patients with protective titers going along with a tendency for reduction in immunosuppressive treatment.

*Conclusions:* Efficacy of vaccination against MenB is insufficient in patients with aHUS. Response to vaccination seems to be hampered by immunosuppression. Therefore, implementation of adequate antibiotic prophylaxis seems pivotal.

## Introduction

Atypical hemolytic uremic syndrome (aHUS) is a rare, but life-threatening systemic disease with an incidence of approximately one to two cases per million [1]. Uncontrolled activation and dysregulation of the complement system in aHUS leads to the development of thrombotic microangiopathy generally characterized by hemolytic anemia, thrombocytopenia, and acute renal failure [2]. Without specific treatment, aHUS often results in end-stage kidney disease [3]. Depending on the underlying complement disorder relapse rates following renal transplantation can approach 80–100% [4,5], putting graft survival at risk [6,7].

The recombinant humanized monoclonal antibody against complement protein C5 eculizumab (Soliris, Alexion Pharmaceuticals), licensed for treatment of paroxysmal nocturnal hemoglobinuria, refractory generalized myasthenia gravis and aHUS in Europe, is the first-line treatment for aHUS [8,9]. Treatment with eculizumab leads to a highly increased risk of infections with capsulated bacteria, especially meningococci [10]. Serogroup B meningococci (MenB) are the most frequently encountered cause for meningococcal infections in Europe [11]. Therefore, vaccination against MenB is recommended in Germany for patients receiving eculizumab and those suffering from innate or acquired immunodeficiencies in addition to vaccination against meningococci serogroups A, C, W, and Y. [12].

While vaccines against serogroups A, C, W, and Y meningococci target the bacterial polysaccharide capsules, vaccines against MenB are directed against outer membrane proteins, because the MenB capsular polysaccharide is not immunogenic and is a potential autoantigen due to its structural identity with the polysaccharide of neural cell adhesion molecules (N-CAM) [13,14]. The multicomponent vaccine Bexsero (GlaxoSmithKline), which is approved in Europe since January 2013, contains four protein-based recombinant antigen components of MenB (4CMenB) which were identified to be particularly immunogenic [15]. Taking into account studies on the expression of vaccine antigens using the meningococcal antigen typing system [14]. It is reasonable to estimate that approximately 82% of the circulating invasive MenB strains in Europe are covered by vaccination with Bexsero [16].

Studies on the efficacy of 4CMenB vaccination are very rare in adult patients. Previous studies have reported general reduced immunogenicity to vaccinations in patients with end-stage renal disease and kidney transplant recipients [17,18]. Immunogenicity of one vaccination with quadrivalent meningococcal polysaccharide conjugate vaccine was insufficient in patients with aHUS [19]. Due to the use of human complement in the bactericidal assay it is currently not possible to analyze serum from patients with ongoing eculizumab treatment, because the complement cascade is blocked by eculizumab [20]. Therefore, studies on the efficacy of 4CMenB vaccination in the already small, but threatened cohort of patients suffering from aHUS are limited to patients currently off treatment with eculizumab and – as to our knowledge – have not been performed.

## Materials and methods

Fourteen patients with aHUS (six males, eight females) born between 1951 and 1994 were vaccinated with two doses of the protein-based serogroup B-vaccine Bexsero according to the recommendations of the manufacturer [21].

A single dose of Bexsero contains 50 mg each of the recombinant proteins: Neisserial heparin-binding antigen (NHBA) fusion protein, adhesin A (NadA) and factor H binding protein (fHbp) fusion protein, and 25 mg outer membrane vesicles (OMVs) derived from meningococcal strain NZ98/254 [21].

Serum bactericidal antibody assay with human complement (hSBA) was performed to measure antibody titers against fHbp as described previously [22,23]. hSBA titers reflect functional antibodies and were calculated as the reciprocal of the final serum dilution killing 50% of bacteria within 1 h. Titers ≥1:4 are regarded to be protective.

Serum samples for hSBA were taken at least 6 weeks following second vaccination, all patients were off treatment with eculizumab at the time. Due to the use of human complement in the bactericidal assay it is currently not possible to analyze serum from patients with eculizumab treatment, because the complement cascade is blocked by eculizumab [20]. Pre-immune serum was not collected routinely.

Differences between means of subgroups were analyzed by two-sided Mann–Whitney test where appropriate. A *P*-value <0.05 was considered to be statistically significant.

The research has been carried out in accordance with the World Medical Association Declaration of Helsinki. Analysis of bactericidal antibody titers and patient data for scientific use was approved by the local ethics committee of the University of Duisburg-Essen (18-8013-BO). In line with the approval of the local ethics committee, due to the retrospective and observational character of the study (vaccinations and measurements of meningococcal antibody titers were performed as part of standard of care) and the fully anonymized analysis of data. Written informed consent was not obtained.

## Results

The cohort consisted of 14 patients previously diagnosed with aHUS (eight females/six males; born between 1951 and 1994). No patient had a history of meningococcal disease or previous meningococcal immunization. In the past, all but one patient had received treatment with eculizumab which was terminated either due to deteriorating kidney function permanently requiring renal replacement therapy without extrarenal disease manifestation or due to treatment discontinuation after recovery and careful risk analysis and monitoring.

If eculizumab treatment was initiated contemporaneously with the first 4CMenB vaccination, patients were additionally vaccinated with a meningococcal quadrivalent conjugate vaccine against serogroups A, C, W, and Y and received at least 2 weeks of appropriate antibiotic prophylaxis, usually with ciprofloxacin, ceftriaxone, or piperacillin/tazobactam depending on additional circumstances. Patients treated with eculizumab before approval of Bexsero in Europe received 4CMenB vaccination after its approval. Eculizumab treatment was ongoing at time of first and second 4CMenB vaccination in 36% of patients, respectively (Table 1).

**Table 1:** Patient characteristics

	At the time of first vaccination					At the time of second vaccination					Titers after second vaccination
Number	Hemodialysis	RTx	Kidney function	Immunosuppressive therapy	Treatment with eculizumab	Hemodialysis	RTx	Kidney function	Immunosuppressive therapy	Treatment with eculizumab	
1	X		RRT	PP + steroids	X	X		RRT		X	Non-protective
2	X		RRT	PP + steroids	X			sCrea 1.4 mg/dl		X	Non-protective
3		X	sCrea 2.4 mg/dl	PP + MMF, tacrolimus, steroids	X		X	sCrea 2.7 mg/dl	MMF, tacrolimus, steroids	X	Non-protective
4		X	sCrea 1.7 mg/dl	MMF, tacrolimus, steroids			X	sCrea 2.0 mg/dl	MMF, tacrolimus, steroids	X	Non-protective
5		X	sCrea 0.9 mg/dl	MMF, belatacept, steroids			X	sCrea 0.8 mg/dl	MMF, belatacept, steroids		Non-protective
6		X	sCrea 1.1 mg/dl	MMF, belatacept, steroids			X	sCrea 1.1 mg/dl	MMF, belatacept, steroids		Non-protective
7		X	sCrea 2.5 mg/dl	MMF, tacrolimus, steroids			X	sCrea 2.2 mg/dl	MMF, tacrolimus, steroids		Non-protective
8			sCrea 4.0 mg/dl	PP + steroids	X	X		RRT		X	Protective
9			sCrea 6.0 mg/dl		X	X		RRT			Protective
10	X		RRT			X		RRT			Protective
11	X		RRT			X		RRT			Protective
12	X		RRT			X		RRT			Protective
13			sCrea 3.6 mg/dl	PP + steroids		X		RRT			Protective
14		X	sCrea 1.1 mg/dl	azathioprine, steroids			X	sCrea 1.1 mg/dl	azathioprine, steroids		Protective
**Protective titers**											**50%**

Vaccination with two doses of the protein-based serogroup B-vaccine Bexsero were performed. Titers <1:4 were regarded to be non-protective. Titer measurements were performed at least 6 weeks after application of second vaccination. *RTx* renal transplant recipient; *RRT* renal replacement therapy; *sCrea* serum creatinine; *PP* plasmapheresis; *MMF* mycophenolmofetil.

Kind of dialysis, kidney function (in patients not on dialysis), treatment with eculizumab and the kind of immunosuppressive therapy at the time of first, respectively second vaccination. Abbreviations: eGFR, estimated glomerular filtration rate; f, female; m, male; MMF, mycophenolate mofetil; PP, plasmapheresis; RRT, renal replacement therapy; RTx, renal transplantation; sCrea, serum creatinine; /, no immunosuppressive therapy.

At time of first vaccination with Bexsero, eleven patients showed compromised kidney function (estimated glomerular filtration rate (eGFR) <60 ml/min) of which five patients had an eGFR ≤30 ml/min and five patients were dependent on renal replacement therapy.

Six patients were kidney transplant recipients receiving immunosuppressive treatment during both vaccinations, while additional three patients received plasmaphereses and steroids for treatment of thrombotic microangiopathy at time of first vaccination only (Table 1).

**Figure 1:**
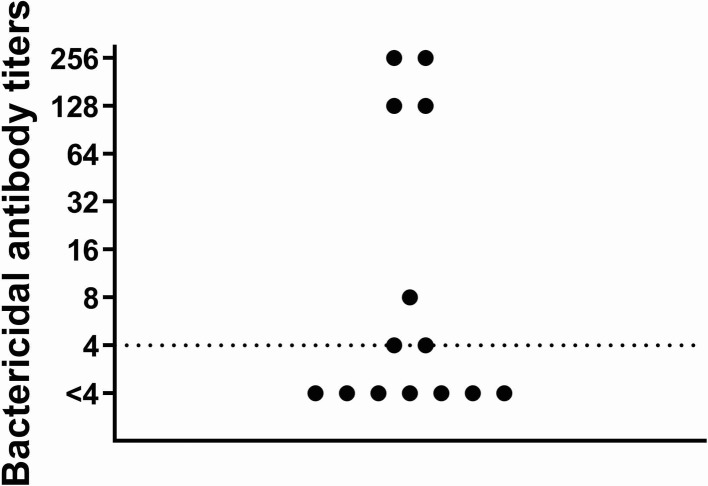
Bactericidal antibody titers against fHbp in patients with aHUS after two doses of Bexsero Serum bactericidal antibody assay with human complement was performed to measure antibody titers against fHbp. hSBA were calculated as the reciprocal of the final serum dilution killing 50% of bacteria. Titers ≥1:4 are regarded to reflect protective antibody response.

Fifty percent of the patients showed protective hSBA titers (≥1:4) against MenB following two vaccinations leaving half of the patients with non-protective titers (Table 1). Bactericidal antibody titers, although regarded to be protective, were relatively low (≤1:8) in three of seven patients with protective antibody titers (Figure 1).

While the majority of our patients (71%) were on immunosuppressive treatment for either thrombotic microangiopathy and/or renal transplantation during either first or second vaccination, alll four patients not receiving any immunosuppressive treatment showed protective bactericidal antibody response.

**Figure 2:**
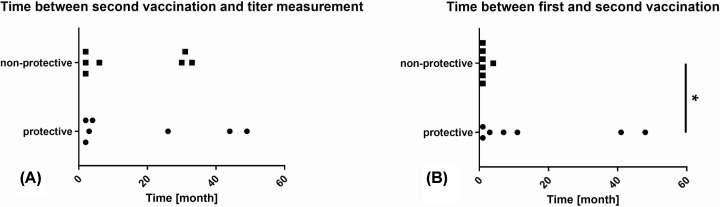
Correlation of time intervals and efficacy of vaccination Correlation of time intervals between second vaccination and titer measurement (**A**) and first and second vaccination (**B**), respectively, with antibody persistence. Time between first and second vaccination varied between 1 and 49 months. Time between first and second vaccination was significantly longer in patients with protective vs. non-protective antibody titers (*P<0.05).

Time between second vaccination and titer measurement varied between 6 weeks and 49 months (median 5 months), but was not significantly different between patients with protective titers compared with those with non-protective titers (Figure 2A). Time between first and second vaccination varied between 1 and 48 months (median 1 month) and was significantly longer in patients with protective bactericidal antibody titers (*P*=0.0338) (Figure 2B).

No obvious impact of ongoing eculizumab treatment or need for renal replacement therapy at the time of vaccination on vaccination response was detected.

## Discussion

Immunogenicity of two doses of 4CMenB vaccination in patients with aHUS was low leaving 50% of patients without protective bactericidal antibody titers.

The estimated risk of invasive meningococcal disease due to serogroup B in patients with innate or acquired complement deficiencies is approaximately 3% per year [24]. Administration of MenB vaccines is therefore strongly recommended in patients receiving eculizumab [12,25,26]. As neither recommendations on the mode of initial immunization nor precise recommendations on re-vaccination are available, immunization was performed with two doses of Bexsero according to the recommendations of the manufacturer [21].

As aHUS is a very acute, kidney and life-threatening disease eculizumab has to be started immediately in symptomatic patients. In contrast to patients with paroxysmal nocturnal hemoglobinuria or myasthenia gravis, eculizumab treatment cannot be delayed to ensure sufficient vaccination as early treatment goes along with improved recovery [27]. If eculizumab treatment is started in parallel to vaccination antibiotic prophylaxis is mandatory during the first 2 weeks of treatment [25,26]. As it remains controversial whether vaccination is effective in patients with acute kidney injury, chronic kidney disease, and/or during immunosuppression [26] – conditions which can be summarized as acquired immunodeficiencies and are common in patients with aHUS – examination of immunogenicity following vaccination is recommended [28]. Nevertheless, antibody titer measurement is currently not covered by statutory health insurance in Germany. Titer measurement in patients with aHUS is hampered by the fact that ongoing eculizumab treatment does currently prohibit titer measurement as the test requires human complement which is blocked by eculizumab [20]. Therefore, it is currently only possible to measure titers in patients off eculizumab treatment limiting the available cohort for antibody titer evaluation. This point is especially relevant for patients previously diagnosed with aHUS who are off treatment due to persisting end-stage renal disease and are on the waiting list for kidney transplantation with expected re-initiation of eculizumab at time of transplantation [29]. These patients are available for both: vaccination without or with minimal immunosuppressive treatment and titer measurements.

Although 4CMenB contains recombinant proteins of NHBA fusion protein, NadA and fHbp fusion protein, and OMV derived from meningococcal strain NZ98/254, according to current practice only antibody titers against fHbp were measured. The protein PorA of OMV used as vaccine antigen can be identified in approximately one-fifth of European MenB strains, NadA is only present in low proportion of MenB strains in Europe and growth conditions normally used for the SBA assay with human complement are not optimal for its expression, while most of European MenB isolates are covered by fHbp and NHBA [16]. As the presence of *fHbp* genes is preserved in nearly all European meningococcal isolated from invasive disease and results of the SBA assay are highly reliable, measurement of SBA against fHbp should reflect the highest proportion of immunogenicity of 4CMenB.

Low pre-existing bactericidal antibodies against meningococci despite vaccination due to asymptomatic nasopharyngeal carriage of *Neisseria meningitidis* in approximately 10% of the general population are a known phenomenon [30], but were not measured in our cohort as they are expected to show only limited response to boostering in immunosuppressed patients [31] and would not have changed the course of action concerning vaccination. In fact, our results of low immunogenicity of two courses of 4CMenB vaccination indicate the insignificance of those pre-existing antibodies in our cohort.

Santolaya et al. [32] reported protective hSBA titers in >90% of healthy adolescents in Chile 1 month after vaccination with either one, two, or three doses of 4CMenB. Protective hBSA titers following two doses of 4CMenB persisted in 77–94% of participants 18–24 months later. These results are in line with Perrett et al. who showed protective hSBA titers against fHbp in 99% of healthy adolescents 1 month after two doses of 4CMenB (1 month apart) [33]. Time between application of the second vaccination and measurement of bactericidal antibody titers was variable. Although, titers are expected to deteriorate after vaccination [32,34] no differences between patients with protective compared with those with non-protective antibody titers were found. Still, booster vaccinations are probably inducing a more robust immune response and are regarded to be safe [34] and should therefore be applied to patients with low or fading antibody titers. Precise recommendations on revaccination as well as on the use of Trumemba (Pfizer), containing two recombinant variants of the surface antigen fHbp (MenB-fHbp) approved in Europe 2017 [35] in patients with low immunological response, are missing.

Vaccine response is generally reduced in patients suffering from reduced kidney function being associated with chronic inflammation and therefore leading to decreased immunity [18,36]. Our cohort included four patients not receiving additional immunosuppressive treatment at time of any vaccination. Those four patients all showed protective bactericidal antibody titers. Despite the small size of our cohort but in line with data for other vaccines [18,37,38], it is reasonable to conclude that immunosuppressive treatment is a major opponent to successful vaccination. This point is also addressed by the fact that the time between first and second vaccination was significantly longer in patients with protective bactericidal antibody response. A longer time interval between vaccinations was associated with a tendency for reduced immunosuppressive treatment. Therefore, application of the second dose of Bexsero should not strictly be carried out 1 month following the first dose, but scheduled individually considering current and planned immunosuppressive treatment. Additionally, evidence for lowering of immunogenicity under immunosuppressive treatment emphasized the advantage of systematic implementation of vaccinations for transplant candidates on the waiting list in order to obtain higher immunization rates which are often neglected despite clear consensus and strong recommendations regarding pre-transplant immunization [39–41].

Antibiotic prophylaxis should be administered in all patients with non-protective or unknown antibody titers against MenB analogous to the procedure after vaccination with the quadrivalent conjugate vaccine against serogroups A, C, W, and Y [19]. There is controversy about the sense of elimination of *Neisseria* sp. in the nasopharynx, e.g. by ciprofloxacin, before down-staging to preferably penicillin V [42]. Applying antibiotic prophylaxis for 2 weeks post-vaccination only as described in the prescribing information of eculizumab [43], is putting patients at risk of meningococcal disease and can therefore not be recommended. In addition, measurable protective antibody titers will decrease the risk of meningococcal disease, but not fully prevent it and protection from vaccination-induced antibodies is questionable under eculizumab-induced C5 blocking by *in vitro* studies [20].

In addition to vaccination under lowest immunosuppressive treatment possible and measurement of bactericidal antibody titers in patients with expected future complement blockade, maintenance of antibiotic prophylaxis for patients on eculizumab treatment and up to 3 months after discontinuation is recommended [26]. Likewise, the patients must be well informed about risk and clinical signs of meningococcal disease and should carry the emergency card if on ongoing eculizumab treatment. Besides maintenance antibiotic treatment patients should be equipped with adequate stand-by antibiotics for break-through infections, while clinicians must even consider meningococcal bacteremia in patients with eculizumab treatment and non-specific symptoms [44].

## Abbreviations

aHUS: atypical hemolytic uremic syndrome
fHbp: factor H binding protein
hSBA: serum bactericidal antibody
MenB: serogroup B meningococci
NadA: Neisserial adhesin A
NHBA: Neisserial heparin-binding antigen
OMV: outer membrane vesicle
4CMenB: four protein-based recombinant antigen components of MenB
